# Predictors of low cervical cancer screening among immigrant women in Ontario, Canada

**DOI:** 10.1186/1472-6874-11-20

**Published:** 2011-05-27

**Authors:** Aisha K Lofters, Rahim Moineddin, Stephen W Hwang, Richard H Glazier

**Affiliations:** 1 Department of Family & Community Medicine, University of Toronto, Toronto, Canada; 2 Department of Family & Community Medicine, St. Michael's Hospital, Toronto, Canada; 3 Centre for Research on Inner City Health, The Keenan Research Centre in the Li Ka Shing Knowledge Institute of St. Michael's Hospital, Toronto, Canada; 4 Primary Health Care System Research Fellow, London, Canada; 5 Institute for Clinical Evaluative Sciences, Toronto, Canada; 6 Dalla Lana School of Public Health, University of Toronto, Toronto, Canada; 7 Department of Medicine, University of Toronto, Toronto, Canada

## Abstract

**Background:**

Disparities in cervical cancer screening are known to exist in Ontario, Canada for foreign-born women. The relative importance of various barriers to screening may vary across ethnic groups. This study aimed to determine how predictors of low cervical cancer screening, reflective of sociodemographics, the health care system, and migration, varied by region of origin for Ontario's immigrant women.

**Methods:**

Using a validated billing code algorithm, we determined the proportion of women who were not screened during the three-year period of 2006-2008 among 455 864 identified immigrant women living in Ontario's urban centres. We created eight identical multivariate Poisson models, stratified by eight regions of origin for immigrant women. In these models, we adjusted for various sociodemographic, health care-related and migration-related variables. We then used the resulting adjusted relative risks to calculate population-attributable fractions for each variable by region of origin.

**Results:**

Region of origin was not a significant source of effect modification for lack of recent cervical cancer screening. Certain variables were significantly associated with lack of screening across all or nearly all world regions. These consisted of not being in the 35-49 year age group, residence in the lowest-income neighbourhoods, not being in a primary care patient enrolment model, a provider from the same region, and not having a female provider. For all women, the highest population-attributable risk was seen for not having a female provider, with values ranging from 16.8% [95% CI 14.6-19.1%] among women from the Middle East and North Africa to 27.4% [95% CI 26.2-28.6%] for women from East Asia and the Pacific.

**Conclusions:**

To increase screening rates across immigrant groups, efforts should be made to ensure that women have access to a regular source of primary care, and ideally access to a female health professional. Efforts should also be made to increase the enrolment of immigrant women in new primary care patient enrolment models.

## Background

Widespread screening using the Papanicolaou (Pap) test has been proven to dramatically reduce cervical cancer rates, and women who develop cervical cancer are most often women who have not been appropriately screened[1-5]. Therefore, in Ontario, Canada, evidence-based guidelines recommend that screening occur at least once every three years for all women with a history of vaginal sexual activity until 70 years of age[5]. However, the literature suggests that adherence to guidelines is not complete or equitable in our setting. Disparities in cervical cancer screening for foreign-born women have long been documented in the Ontario and Canadian literature, using both self-report and administrative data[6-13]. This risk of non-screening is not equal across immigrant groups. In our previous work, although all immigrant groups had significantly lower screening rates than long-term residents of the province [53.1% vs. 64.6%), women from South Asia and from the Middle East and North Africa were the most vulnerable to lack of screening, and women from Western Europe and from Latin America and the Caribbean were the least vulnerable[14]. These findings were in the context of screening rates and knowledge about the Pap test being quite low in many South Asian and Middle Eastern countries. [2,15,16].

The underlying mechanisms for screening disparities for immigrant women may lie in sociodemographic barriers, barriers rooted in the health care system, cultural or migration-related barriers or, most likely, some combination of the three [4,17-23]. As well, it is feasible that the relative importance of these barriers varies between ethnic groups. For example, the gender of the physician performing the Pap test or language barriers may be of more importance for women from one cultural or language group than from another. Therefore, the aims of this study were to: i) determine if the independent effects on cervical cancer screening of various factors reflective of sociodemographics, the health care system, and culture and migration were modified by region of origin for identified immigrant women in Ontario, and ii) to calculate population-attributable fractions for these factors for each region of origin.

## Methods

### Study Setting

According to the 2006 Census, Ontario is Canada's largest province with a population of over 12 million people, over 28% of whom are foreign-born[24]. More than half of all Canada's immigrants settle in Ontario[25]. Asia is currently the main source continent, and India the number one source country, for newcomers to the province[25]. Nearly 75% of the province's population live in one of fifteen census metropolitan areas (CMAs) i.e. a geographic area with a total population of at least 100 000, of which 50 000 or more live in an urban core[26,27]. For Ontario's foreign-born population, 94.0% live in a CMA[28]. Therefore, we limited the study setting to Ontario's CMAs. Ontario has a single, government-run, universal health insurance plan that pays for all medically necessary services, including cervical cancer screening.

### Data Sources

Details on data sources have been previously published elsewhere[14].

### Study Cohort

Our cohort consisted of all women in Ontario who were alive and eligible for health coverage from January 1, 2006 to December 31, 2008, who ranged in age from 18 to 69 years for the entire three-year study period, whose most recent postal code was in a CMA, and who were identified immigrants based on the Landed Immigrant Data System (LIDS). A total of 524 997 women fit these initial inclusion criteria.

To ensure that we captured Pap tests performed for screening and not diagnostic purposes, we excluded women with an available history of gynaecological cancer in Ontario Cancer Registry records (1 427 women), or colposcopy in physicians' claims records (42 704 women). Women who have had a total hysterectomy are no longer screening-eligible, therefore we also excluded 26 598 women with an available history of such in hospital discharge records. Ontario has newly instituted primary care patient enrolment models (PEMs), which include financial incentives for family physicians to perform cervical cancer screening on enrolled women aged 35-69 years. Because the tracking code Q140A can be claimed by Ontario physicians who participate in these models for any enrolled female patient aged 35-69 years who is ineligible for cervical cancer screening for any reason, we excluded 6 008 women who had a Q140A code claimed at least once in available records. Due to overlap of reasons for exclusion, a total of 69 133 women were excluded. Therefore, the final cohort consisted of 455 864 women.

### Stratified Multivariate Analysis

To classify women by region of origin, we accessed LIDS to determine country of birth for each woman. The countries were then grouped into regions based on a modification of the classification system used by the World Bank[29]. To determine if region of origin acted as an effect modifier, we conducted a stratified multivariate analysis by creating eight identical models, stratified by the eight world regions.

As our outcome is relatively common, odds ratios determined from logistic regression would not provide an accurate estimate of relative risks. Therefore, we used multivariate Poisson regression[30] to estimate adjusted relative risks. Models included variables in three categories: sociodemographic, health care-related and migration-related variables that may influence the likelihood of cervical cancer screening. For sociodemographic variables, we considered:

• age category (18-34 years, 35-49 years [referent], 50-66 years) as there are financial incentives in PEMs for screening women 35 years and over and as women 50 years and over have previously been found to be under-screened[6,8,31]

• neighbourhood income quintile

• whether each woman lived in a small urban versus large urban setting based on her Rurality Index of Ontario score

• whether she had a university degree at the time of landing in Ontario. Health care-related variables included:

• whether each woman had at least one major prenatal visit during the study period, as Pap tests are expected aspects of these visit types and may be more acceptable during these visits

• whether she had seen a gynaecologist at least once during the study period

• whether she was rostered in a PEM

• whether she was virtually rostered i.e. saw a family physician regularly who participates in a PEM despite the woman not being enrolled herself

• whether the woman had at least one female provider (either family physician or gynaecologist)

• whether she had at least one provider from the same region of the world (either family physician or gynaecologist) based on the physician's medical school

• the presence of co-morbidities in the two years prior to the index date based on the Johns Hopkins Adjusted Clinical Groups Case-Mix System, which. uses diagnostic information from administrative databases to describe and predict patients' use of health care resources. In this study, we used Resource Utilization Bands, which range from 0 (lowest expected health care costs) to 5 (highest expected health care costs), to categorize patients based on their expected use of health care resources, and Aggregated Diagnosis Groups, which range from 0 (no diagnosis group) to 32 (a maximum of 32 distinct diagnosis groups) to categorize the level of co-morbidity. This system has been validated for use in Canadian populations[32].

Migration-related variables included:

• immigrant class (economic [referent], family, refugee)

• English speaking ability at landing

• whether she had been in Canada less than 10 years

• age at landing.

SAS Version 9.1 (SAS Institute Inc, Cary, NC) was used to fit all models and determine adjusted relative risks.

Adjusted relative risks (ARRs) were then used to calculate population-attributable fractions (PAFs) for each variable using the following formula:

where *p *was the proportion of the study population with the variable of interest.

### Outcome Definition

To determine if each woman in the cohort had been appropriately screened for cervical cancer, we used a previously validated billing code-based algorithm consisting of all procedural codes that can be billed by the physician performing the Pap test and all laboratory codes that can be billed by the cytopathologist interpreting the Pap test. A woman was considered appropriately screened if at least one of the specified billing codes had been claimed for her in the three-year study period. This algorithm had 99.5% sensitivity and 85.7% specificity when compared to a Pap test registry [6].

## Results

Characteristics of the study population are summarized in Table 1. The largest immigrant groups were from East Asia and the Pacific (128 965 women) and from South Asia (88 107 women). The smallest group was from the USA, Australia and New Zealand (10 003 women). Women from Latin America and the Caribbean and from Sub-Saharan Africa were most likely to be living in the poorest neighbourhoods, and least likely to have a university degree. There was a mismatch between university-level education and income for South Asian women, with a high representation in low-income neighbourhoods but a high level of educational attainment. Women from the USA, Australia and New Zealand had the highest educational attainment, and had the least amount of health care contact. South Asian women were the most likely to have at least one female provider and to have a provider from the same region of the world, and were most commonly rostered in PEMs. Eastern European and Central Asian women were the least likely to be able to speak English at landing, and women from Sub-Saharan Africa were the most likely to arrive as refugees.

**Table 1:** Demographic characteristics of the 455 864 identified immigrant women in the cohort who were aged 18-66 on January 1, 2006 by region of origin.

	East Asia &Pacific	Eastern Europe & Central Asia	Latin America & Caribbean	Middle East & North Africa	South Asia	Sub-Saharan Africa	USA, Australia & New Zealand	Western Europe	All identified Immigrants
**n**	128 965	67 845	70 184	33 649	88 107	26 125	10 003	30 167	455 864

**SOCIODEMOGRAPHIC FACTORS**									

**Mean age (SD)**	40.6 (11.3)	39.5 (11.8)	38.8 (11.8)	37.2 (12.1)	38.6 (11.8)	37.1 (10.9)	39.7 (11.8)	38.7 (11.5)	39.2 (11.7)

**Age category, No. (%):**									
**18-34 years**	37 321 (28.9)	22 413 (33.0)	25 852 (36.8)	14 333 (42.6)	35 565 (40.4)	10 428 (39.9)	3 043 (30.4)	10 382 (34.4)	159 581 (35.0)
**35-49 years**	63 857 (49.5)	3 137 (45.9)	30 936 (44.1)	13 743 (40.8)	35 904 (40.8)	12 377 (47.4)	4 903 (49.0)	14 596 (48.4)	207 836 (45.6)
**50-66 years**	27 787 (21.6)	14 295 (21.1)	13 396 (19.1)	5 573 (16.6)	16 638 (18.9)	3 320 (12.7)	2 057 (20.6)	5 189 (17.2)	88 447 (19.4)

**Income Quintile, No. (%)**									
**Q1 (lowest)**	31 755 (24.6)	17 591 (25.9)	25 352 (36.1)	9 045 (26.9)	27 878 (31.6)	11 908 (45.6)	1 439 (14.4)	5 730 (19.0)	130 867 (28.7)
**Q2**	32 387 (25.1)	13 136 (19.4)	17 696 (25.2)	6 402 (19.0)	23 281 (26.4)	5 042 (19.3)	1 591 (15.9)	6 788 (22.5)	106 489 (23.4)
**Q3**	25 862 (20.1)	13 403 (19.8)	13 668 (19.5)	6 511 (19.4)	19 038 (21.6)	3 629 (13.9)	1 692 (16.9)	5 807 (19.3)	89 798 (19.7)
**Q4**	22 197 (17.2)	14 215 (21.0)	8 518 (12.1)	6 537 (19.4)	11 853 (13.5)	2 985 (11.4)	1 948 (19.5)	5 800 (19.2)	74 218 (16.3)
**Q5 (highest)**	16 556 (12.8)	9 434 (13.9)	4 841 (6.9)	5 105 (15.2)	5 964 (6.8)	2 478 (9.5)	3 311 (33.1)	5 997 (19.9)	53 815 (11.8)

No. (%) with university degree*	28 369 (22.0)	16 729 (24.7)	4 029 (5.7)	6 832 (20.3)	21 923 (24.9)	2 317 (8.9)	3 390 (33.9)	2 740 (9.1)	86 525 (19.0)

**No. (%) living in large urban area**	124 405 (96.5)	62 988 (92.9)	66 975 (95.5)	31 971 (95.1)	85 190 (96.7)	25 221 (96.6)	8 127 (81.4)	26 142 (86.7)	431 805 (94.8)

**HEALTH CARE-RELATED FACTORS**									

**No. (%) in RUB category:**									
**0-1**	34 440 (26.7)	13 368 (19.7)	10 967 (15.6)	8 170 (24.3)	13 835 (15.7)	5 591 (21.4)	4 592 (45.9)	10 138 (33.6)	101 345 (22.2)
**2**	19 351 (15.0)	10 843 (16.0)	8 439 (12.0	3 946 (11.7)	10 419 (11.8)	3 048 (11.7)	1 210 (12.1)	3 989 (13.2)	61 365 (13.5)
**3**	57 936 (44.9)	33 743 (49.7)	36 782 (52.4)	15 170 (45.1)	44 120 (50.1)	11 791 (45.1)	3 154 (31.5)	12 118 (40.2)	215 149 (47.2)
**4-5**	17 238 (13.4)	9 891 (14.6)	13 996 (19.9)	6 363 (18.9)	19 733 (22.4)	5 695 (21.8)	1 047 (10.5)	3 922 (13.0)	78 005 (17.1)

**No. (%) in ADG category:**									
**0-5**	83 780 (65.0)	42 490 (62.6)	37 137 (52.9)	19 009 (56.5)	44 845 (50.9)	14 426 (55.2)	21 046 (69.8)	21 046 (69.8)	270 893 (59.4)
**6-9**	35 926 (27.9)	20 473 (30.2)	24 909 (35.5)	10 527 (31.3)	32 035 (36.4)	8 542 (32.7)	7 300 (24.2)	7 300 (24.2)	141 848 (31.1)
**10+**	9 259 (7.2)	4 882 (7.2)	8 138 (11.6)	4 113 (12.2)	11 227 (12.7)	3 157 (12.1)	1 821 (6.0)	1 821 (6.0)	43 123 (9.5)

**No. (%) with prenatal visit during study period**	5 736 (4.5)	3 579 (5.3)	4 610 (6.6)	1 905 (5.7)	7 312 (8.3)	2 000 (7.7)	321 (3.2)	1 507 (5.0)	26 995 (5.9)

**No. (%) in: Patient enrolment model**	80 584 (62.5)	41 953 (61.8)	47 620 (67.9)	21 227 (63.1)	63 195 (71.7)	16 682 (63.9)	5 101 (51.0)	17 707 (58.7)	294 553 (64.6)
**Virtually rostered**	23 706 (18.4)	17 131 (25.3)	14 979 (21.3)	6 302 (18.7)	15 499 (17.6)	5 374 (20.6)	1 123 (11.2)	4 482 (14.9)	88 732 (19.5)
**Neither**	24 675 (19.1)	8 761 (12.9)	7 585 (10.8)	6 120 (18.2)	9 413 (10.7)	4 069 (15.6)	3 779 (37.8)	7 978 (26.5)	72 579 (15.9)

**No. (%) with female provider**	45 676 (35.4)	28 917 (42.6)	23 234 (33.1)	12 555 (37.3)	42 068 (47.8)	9 679 (37.1)	3 188 (31.9)	10 866 (36.0)	176 471 (38.7)

**No. (%) with provider from same region**	28 081 (21.8)	19 713 (29.1)	5 397 (7.7)	8 380 (24.9)	34 374 (39.0)	1 937 (7.4)	58 (0.6)	2 911 (9.7)	100 851 (22.1)

**No. (%) with gynaecologist**	27 739 (21.5)	20 547 (30.3)	22 028 (31.4)	8 822 (26.2)	23 768 (27.0)	7 557 (28.9)	1 472 (14.7)	5 985 (19.8)	118 075 (25.9)

**MIGRATION-RELATED FACTORS**									

No. (%) able to speak English*	73 105 (56.7)	24 623 (36.3)	57 012 (81.2)	17 673 (52.5)	43 638 (49.5)	18 700 (71.6)	9 675 (96.7)	19 901 (66.0)	264 848 (58.1)

**No. (%) in Canada less than 10 yrs**	18 931 (14.7)	10 919 (16.1)	6 184 (8.8)	5 578 (16.6)	20 179 (22.9)	3 700 (14.2)	1 094 (10.9)	1 908 (6.3)	68 827 (15.1)

**Mean age at landing (SD)**	28.8 (11.5)	27.8 (12.0)	25.5 (12.0)	25.8 (12.4)	28.1 (11.9)	25.2 (11.0)	26.3 (12.1)	24.2 (11.9)	27.2 (11.9)

**Immigrant class, No. (%)**									
**Economic**	74 615 (57.9)	26 577 (39.2)	21 684 (30.9)	16 956 (50.4)	30 904 (35.1)	7 669 (29.4)	3 508 (35.1)	19 438 (64.4)	201 872 (44.3)
**Family**	47 632 (36.7)	21 099 (31.1)	41 312 (58.9)	8 688 (25.8)	43 243 (49.1)	8 450 (32.3)	6 255 (62.5)	9 783 (32.4)	186 444 (40.9)
**Refugee**	5 817 (4.5)	19 621 (28.9)	7 010 (10.0)	7 836 (23.3)	13 278 (15.1)	9 834 (37.6)	238 (2.4)	940 (3.1)	64 618 (14.2)

*Recorded on date of landing in Canada

RUB = Resource Utilization Bands, which range from 0 (lowest expected health care costs) to 5 (highest expected health care costs), used to categorize patients based on their expected use of health care resources.

ADG = Aggregated Diagnosis Groups, which range from 0 (no diagnosis group) to 32 (32 distinct diagnosis groups) used to measure the level of co-morbidity.

A total of 213 729 women (46.9%) were not screened for cervical cancer during the three-year study period. Table 2 displays numbers and percentages of women who were not recently screened by region of origin for particular variables of interest. Women who had at least one female provider were the least likely to be unscreened, with the lowest number of unscreened women seen among Caribbean and Latin American women who had at least one female provider (21.2%). The highest proportions of unscreened women were seen among those women who were neither in a PEM nor virtually rostered, with percentages consistently above 90%. Among those women who were neither in a PEM nor virtually rostered, only 11.8% had any contact at all with the health care system during the study period (i.e. a physician office visit, hospitalization, emergency room visit, laboratory test, imaging procedure, or drug benefit claim). Of these women who had health care contact, 70.1% were still not recentlyly screened, ranging from 61.1% for women from the USA, Australia and New Zealand to 77.4% for Middle East and North African women (data not shown).

**Table 2:** Number (and percentage) of women without a Pap test in 2006-8 among the 455 864 identified immigrant women in the cohort who were aged 18-66 on January 1, 2006 by region of origin.

	East Asia & Pacific	Eastern Europe & Central Asia	Latin America & Caribbean	Middle East & North Africa	South Asia	Sub-Saharan Africa	USA, Australia & New Zealand	Western Europe	All identified immigrants
**SOCIODEMOGRAPHIC FA CTORS**									

**Age category:**									
**18-34 years**	19 513 (52.3)	8 640 (38.6)	9 305 (36.0)	8 082 (56.4)	17 828 (50.1)	5 443 (52.2)	1 913 (62.9)	4 991 (48.1)	75 863 (47.5)
**35-49 years**	28 916 (45.3)	12 327 (39.6)'	10 214 (33.0)	6 358 (46.3)	15 689 (43.7)	5 643 (45.6)	2 670(54.5)	7 009 (48.0)	89 019 (42.8)
**50-66 years**	15 053 (54.2)	7 456 (52.2	6 412 (47.9)	3 146 (56.5)	10 503 (63.1)	1 768 (53.3)	1 344 (65.3)	3 057 (58.9)	48 847 (55.2)
									
**Income Quintile**									
**Q1 (lowest)**	16 980 (53.5)	9 102 (51.7)	10 078 (39.8)	5 375 (59.4)	15 358 (55.1)	6 494 (54.5)	976 (67.8)	3 330 (58.1)	67 778 (51.8)
**Q2**	15 677 (48.4)	5 827 (44.4)	6 612 (37.4)	3 472 (54.2)	11 534 (49.5)	2 405 (47.7)	985 (61.9)	3 522 (51.9)	50 126 (47.1
**Q3**	11 974 (46.3)	5 078 (37.9)	4 519 (33.1)	3 103 (47.7)	8 794 (46.2)	1 658 (45.7)	972 (57.5)	2 750 (47.4)	38 957 (43.4)
**Q4**	10 279 (46.3)	4 933 (34.7)	2 839 (33.3)	3 112 (47.6)	5 445 (45.9)	1 225 (41.0)	1063 (54.6)	2 580 (44.5)	31 565 (42.5)
**Q5 (highest)**	8 443 (51.0)	3 441 (36.5)	1 832 (37.8)	2 492 (48.8)	2 838 (47.6)	1 017 (41.0)	1 913 (57.8)	2 845 (47.4)	24 893 (46.3)
									
**University degree:**									
**Yes**	14 141 (49.9)	6 605 (39.5)	1 661 (41.2)	3 495 (51.2)	10 426 (45.1)	1 046 (45.1)	1 815 (53.5)	1 349 (49.2)	40 665 (47.0)
**No**	49 341 (49.1)	21 818 (42.7)	24 270 (36.7)	14 091 (52.6)	33 594 (50.8)	11 808 (49.6)	4 112 (62.2)	13 708 (50.0)	173 064 (46.9)

**HEALTH CARE-RELATED FACTORS**									

**Patient enrolment model**	29 196 (36.2)	13 403 (32.0)	13 366 (28.1)	8 545 (40.3)	27 200 (43.0)	6 358 (38.1)	1 770 (34.7)	5 512(31.1)	105 538 (35.8)
**Virtually rostered**	10 262 (43.3)	6 803 (39.7)	5 465 (36.5)	3 072 (48.8)	7 707 (49.7)	2 594 (48.3)	450 (40.1)	1 760 (39.3)	38 177 (43.0)
**Neither**	24 024 (97.4)	8 217 (93.8)	7 100 (93.6)	5 969 (97.5)	9 113 (96.8)	3 902 (95.9)	3 707 (98.1)	7 785 (97.6)	70 014 (96.5)
									
**Female provider:**									
**Yes**	11 434 (25.0)	7 359 (25.5)	4 924 (21.2)	3 971 (31.6)	13 947 (33.2)	2 961 (30.6)	873 (27.4)	2 653 (24.4)	48 220 (27.3)
**No**	52 048 (62.5)	21 064 (54.1)	21 007 (44.7)	13 615 (64.5)	30 073 (65.3)	9 893 (60.2)	5 054 (74.2)	12 404 (64.3)	165 509 (59.2)
									
**region:**									
**Yes**	11 279 (40.2)	6 474 (32.8)	1 546 (28.7)	3 653 (43.6)	14 760 (42.9)	614 (31.7)	19 (32.8)	992 (34.1)	39 337 (39.0)
**No**	52 203 (51.8)	21 949 (45.6)	24 385 (37.6)	13 933 (55.1)	29 260 (54.5)	12 240 (50.6)	5 908 (59.4)	14 065 (51.6)	174 392 (49.1)

**MIGRATION-RELATED F ACTORS**									

**Able to speak English:**									
**Yes**	36 886 (50.5)	10 012 (40.7)	20 479 (35.9)	9 165 (51.9)	21 656 (49.6)	8 974 (48.0)	5 718 (59.1)	9 867 (49.6)	123 036 (46.5)
**No**	26 596 (47.6)	18 411 (42.6)	5 452 (41.4)	8 421 (52.7)	22 364 (50.3)	3 880 (52.3)	209 (63.7)	5 190 (50.6)	90 693 (47.5)
									
**In Canada:**									
**less than 10 years**	8 055 (42.6)	4 381 (40.1)	2 049 (33.1)	2 840 (50.9)	10 272 (50.9)	1 744 (47.1)	541 (49.5)	851 (44.6)	30 930 (44.9)
**10+ years**	55 427 (50.4)	24 042 (42.2)	23 882 (37.3)	14 746 (52.5)	33 748 (49.7)	11 110 (49.5)	5 386 (60.5)	14 206 (50.3)	182 799 (47.2)
**Immigrant class:**									
**Economic**	39 334 (52.7)	10 684 (40.2)	8 599 (39.7))	9 589 (56.6)	16 810 (54.4)	3 465 (45.2)	2 395 (68.3)	9 995 (51.4)	101 173 (50.1)
**Family**	21 369 (45.1)	9 060 (42.9)	14 442 (35.0)	4 150 (47.8)	20 514 (47.4)	3 821 (45.2)	3 396 (54.3)	4 584 (46.9)	81 461 (43.7)
**Refugee**	2 412 (41.5)	8 451(43.1)	2 822 (40.3)	3 761 (48.0)	6 357 (47.9)	5 499 (55.9)	135 (56.7)	475 (50.5)	29 932 (46.3)
									
**TOTAL WITHOUT A PAP TEST IN 2006-8**	63 482 (49.2)	28 423 (41.9)	25 931 (36.9)	17 586 (52.3)	44 020 (50.0)	12 854 (49.2)	5 927 (59.2)	15 057 (49.9)	213 729 (46.9)

In our eight stratified models, there was little effect modification by region (Table 3). Certain variables were significantly associated with lack of screening across all or nearly all world regions. These consisted of being in either the youngest or oldest age groups and in the lowest income quintiles among the sociodemographic variables; and not being in a patient enrolment model, having a provider from the same region, and not having a female provider among the health care-related variables. None of the migration-related variables were consistently significantly associated with lack of screening. Being unable to speak English at landing trended toward increased risk for most women, but equated to significantly decreased risk for East Asian and Pacific women. Immigrant class was only significant for Sub-Saharan African women and Western European women, with refugees being at higher risk of non-screening in these two groups. Post-hoc, we tested for an interaction between female provider and provider from the same region of the world, but it was not pervasively statistically significant so we did not include this interaction in the models.

**Table 3:** Adjusted relative risks [with 95% confidence intervals] for risk of non-screening for the 455 864 identified immigrant women in the cohort who were aged 18-66 on January 1, 2006 by region of origin.

	East Asia & Pacific	Eastern Europe & Central Asia	Latin America & Caribbean	Middle East & North Africa	South Asia	Sub-Saharan Africa	USA, Australia & New Zealand	Western Europe	All identified immigrants
**SOCIODEMOGRAPHIC FACTORS**									

**Age category:**									
**18-34 years**	1.20 [1.16-1.23]	1.16 [1.11-1.21]	1.22 [1.17-1.27]	1.19 [1.13-1.26]	1.21 [1.17-1.25]	1.16 [1.10-1.24]	1.13 [1.03-1.25]	1.19 [1.12-1.27]	1.24 [1.22-1.26]
**35-49 years**	1.0	1.0	1.0	1.0	1.0	1.0	1.0	1.0	1.0
**50-66 years**	1.20 [1.17-1.24]	1.08 [1.04-1.13]	1.24 [1.19-1.30]	1.16 [1.10-1.23]	1.30 [1.25-1.35]	1.09 [1.02-1.17]	1.06 [0.97-1.16]	1.10 [1.04-1.17]	1.15 [1.13-1.17]
									
**Q1 (lowest)**	1.10 [1.08-1.13]	1.15 [1.11-1.20]	1.10 [1.05-1.16]	1.14 [1.08-1.19]	1.12 [1.08-1.17]	1.21 [1.13-1.30]	1.09 [1.00-1.18]	1.12 [1.06-1.18]	1.14 [1.12-1.15]
**Q2**	1.03 [1.01-1.06]	1.09 [1.05-1.14]	1.06 [1.00-1.11]	1.09 [1.03-1.15]	1.07 [1.03-1.11]	1.12 [1.04-1.20]	1.06 [0.98-1.15]	1.09 [1.03-1.14]	1.07 [1.06-1.09]
**Q3**	1.01 [0.98-1.04]	1.03 [0.99-1.08]	0.97 [0.92-1.03]	1.01 [0.96-1.07]	1.02 [0.98-1.07]	1.12 [1.03-1.21]	1.03 [0.95-1.11]	1.06 [1.01-1.12]	1.03 [1.01-1.04]
**Q4**	0.99 [0.96-1.02]	1.00 [0.95-1.04]	0.97 [0.91-1.03]	1.01 [0.96-1.07]	1.00 [0.96-1.05]	1.01 [0.93-1.10]	0.99 [0.91-1.06]	1.00 [0.95-1.06]	1.0 [0.98-1.01]
**Q5 (highest)**	1.0	1.0	1.0	1.0	1.0	1.0	1.0	1.0	1.0
									
**University degree:**									
**No**	0.98 [0.96-1.00]	1.10 [1.07-1.13]	1.05 [0.99-1.10]	1.01 [0.97-1.05]	1.06 [1.03-1.08]	1.03 [0.96-1.10]	1.06 [1.00-1.13]	1.04 [0.98-1.11]	1.01 [1.00-1.02]
**Yes**	1.0	1.0	1.0	1.0	1.0	1.0	1.0	1.0	1.0

**HEALTH CARE-RELATED FACTORS**									
**No family doctor**	1.33 [1.30-1.36]	1.47 [1.41-1.52]	1.56 [1.49-1.62]	1.31 [1.24-1.37]	1.21 [1.17-1.26]	1.31 [1.24-1.39]	1.28 [1.17-1.39]	1.52 [1.44-1.60]	1.39 [1.37-1.41]
**Virtually rostered**	1.19 [1.16-1.21]	1.23 [1.19-1.27]	1.28 [1.24-1.32]	1.18 [1.13-1.23]	1.13 [1.10-1.16]	1.21 [1.15-1.26]	1.17 [1.05-1.29]	1.26 [1.20-1.33]	1.18 [1.17-1.20]
**Patient enrolment model**	1.0	1.0	1.0	1.0	1.0	1.0	1.0	1.0	1.0
									
**Female provider:**									
**No**	1.58 [1.55-1.62]	1.44 [1.40-1.49]	1.40 [1.35-1.45]	1.32 [1.27-1.38]	1.44 [1.41-1.47]	1.33 [1.27-1.39]	1.43 [1.31-1.55]	1.44 [1.38-1.52]	1.43 [1.41-1.45]
**Yes**	1.0	1.0	1.0	1.0	1.0	1.0	1.0	1.0	1.0
									
**Provider from same region:**									
**Yes**	1.06 [1.04-1.09]	1.08 [1.05-1.12]	1.08 [1.02-1.13]	1.13 [1.08-1.17]	1.09 [1.07-1.11]	0.94 [0.86-1.02]	0.98 [0.62-1.53]	1.14 [1.07-1.22]	1.15 [1.13-1.16]
**No**	1.0	1.0	1.0	1.0	1.0	1.0	1.0	1.0	1.0

**MIGRATION-RELATED FACTORS**									
**Able to speak English**									
**No**	0.98 [0.96-0.99]	1.01 [0.98-1.04]	1.04 [1.01-1.08]	1.04 [1.00-1.07]	1.09 [1.06-1.11]	1.06 [1.02-1.10]	1.05 [0.90-1.22]	1.03 [1.00-1.07]	1.04 [1.03-1.05]
**Yes**	1.0	1.0	1.0	1.0	1.0	1.0	1.0	1.0	1.0
									
**In Canada:**									
**less than 10 years**	1.00 [0.98-1.03]	1.01 [0.98-1.05]	0.97 [0.92-1.02]	1.06 [1.02-1.11]	1.09 [1.06-1.11]	1.05 [0.99-1.11]	1.10 [1.00-1.22]	1.05 [0.97-1.13]	1.05 [1.04-1.07]
**10+ years**	1.0	1.0	1.0	1.0	1.0	1.0	1.0	1.0	1.0
									
**Immigrant class:**									
**Family**	1.00 [0.99-1.02]	1.01 [0.98-1.04]	0.98 [0.95-1.01]	0.96 [0.93-1.00]	0.98 [0.95-1.00]	1.05 [1.00-1.10]	0.97 [0.91-1.02]	0.98 [0.95-1.02]	0.99 [0.98-1.00]
**Refugee**	0.96 [0.92-1.00]	1.00 [0.97-1.03]	1.03 [0.99-1.08]	1.00 [0.96-1.04]	1.02 [0.99-1.05]	1.20 [1.15-1.26]	1.07 [0.89-1.29]	1.08 [0.98-1.18]	1.06 [1.04-1.07]
**Economic**	1.0	1.0	1.0	1.0	1.0	1.0	1.0	1.0	1.0

Relative risks adjusted for all variables listed in the table.

We then determined PAFs for these variables (data not shown). For all women, the highest PAFs were seen for not having a female provider, with values ranging from 16.8% [95% CI 14.6-19.1%] among women from the Middle East and North Africa to 27.4% [95% CI 26.2-28.6%] for women from East Asia and the Pacific. The next highest PAFs varied by region of origin. Risk of non-screening could be attributed to being in the youngest age group for Latin American and Caribbean women (7.4% [95% CI 5.7-9.1%]), Middle Eastern and North African women (7.5% [95% CI 5.1-9.9%]), and South Asian women (7.7% [95% CI 6.4-9.1%]). Being neither rostered nor virtually rostered in a primary care model was of especial importance for women from East Asia and the Pacific (5.9% [95% CI 5.3-6.5%]), the USA, Australia and New Zealand (9.5% [95% CI 6.2-12.8%]), and Western Europe (12.1% [95% CI 10.4-13.8%]), as was not having a university degree for Eastern European and Central Asian women (6.9% [95% CI 4.7-9.1%]), and being in the lowest income quintile for Sub-Saharan African women (8.9% [95% CI 5.7-12.1%]).

## Discussion

The cervical cancer screening rate of 53.1% that we have demonstrated for a three-year period for Ontario's immigrant women living in urban areas, all of whom were eligible for the provincial universal health care system, is substantially lower than would be expected with adherence to provincial guidelines[5], and substantially lower than the 64.6% we have previously found for long-term residents of Ontario living in urban areas during the same time period[14]. Sociodemographic and health care-related factors, namely living in the lowest-income neighbourhoods, not being in the 35-49 year age group, not being either rostered or virtually rostered in a patient enrolment model, and having either a male family doctor or a family doctor from the same region of the world were independently associated with lower rates of screening for immigrant women across most or all regions of origin, suggesting that these variables tend to negatively affect screening for immigrant women regardless of their culture or ethnicity. Even when limiting to women with at least one contact with the health care system during the study period, the prevalence of non-screening was still quite high for women without a family doctor, suggesting that complete lack of health care system contact did not explain this finding.

Our findings are similar to those of other studies that have shown that the gender and cultural origin of the family physician, and income and age of the patient, matter for cervical cancer screening. In another Canadian study, Decker et al. demonstrated that Canadian medical graduates and female physicians were more likely than international medical graduates and male physicians respectively to perform Pap tests[33]. In their literature review, Akers et al. noted that female doctors were consistently more likely to perform cervical screening and that having a doctor of the same ethnicity was associated with lower rates of screening[34]. Tu et al. showed that female physicians were more likely to screen for breast and cervical cancer among Chinese immigrants in both Seattle, USA and Vancouver, Canada[35], and in qualitative studies, immigrant women consistently report that having a female perform the Pap test would increase their comfort level[36-40]. Low-income women have frequently been highlighted as vulnerable to under-screening, both among foreign-born women and among the general population[2,6-8,23,33,41,42]. Although many international studies have shown that older age is associated with lower rates of cervical cancer screening among both immigrants and the general population[2,3,8,40,43-49], only a few studies have highlighted women in the youngest age group as vulnerable to under-screening and most of these have focussed on women younger than 25 years[23,49-51]. It must be noted that the benefits of screening for women under 25 years may be limited[52].

We also used PAFs to determine the screening barriers of most importance for each cultural group, and found that access to a female provider had the highest attributable risk across regions of origin. Other characteristics which decision makers could focus on were also highlighted with some differences across regions. These findings can be used for screening interventions that are targeted at particular ethnic groups. For example, researchers and policymakers aiming to increase screening among Sub-Saharan African women may wish to focus their efforts on women living in the poorest neighbourhoods.

### Strengths and Limitations

This study has several strengths. It is a large, population-based study with broad inclusion criteria that distinguishes immigrant women from all major geographic regions of the world. It uses a previously validated outcome measure[6] instead of self-report to document cervical cancer screening. Self-report is known to systematically overestimate screening attendance[53,54]., It also relies on objective data instead of self-reported data for immigration status and region of origin. The effects of sociodemographics, health care-related factors and migration-related factors on screening for immigrant women from all regions of the world were considered. As well, this is the only study that we are aware of that has examined region of origin as a potential source of effect modification and calculated region-specific population-attributable fractions in order to determine barriers to Pap test use of the most importance for each cultural group.

This study also has several limitations. First, not all potentially relevant information, such as religion, is available from administrative data. Second, some data were only available for women at the time of landing, such as education attainment and language ability. These may have changed for many women by the beginning of the study period. Third, identified immigrants were classified based on their country of birth, which may not always reflect their ethnic and cultural origins. For example, 238 women born in the U.S. were in the refugee class, most likely reflective of women of other ethnic origins whose families lived for a time in the U.S. before settling in Canada. Fourth, although we excluded women where there was any evidence of a hysterectomy in available data, we could not identify out-of-province hysterectomies, which may be relatively common among the immigrant women in the cohort. However, we also excluded women who had a Q140 code billed, which allows physicians in primary care enrolment models to flag those patients whom they have deemed ineligible for screening. All identified immigrants had been in Ontario for over five years by the first day of the study period, and on average, had been in the province for over 10 years, decreasing the likelihood of out-of-province hysterectomies. Finally, our results may not be generalizable to other settings, either inside or outside Canada, as other settings may have different immigrant demographic profiles.

## Conclusions

Our findings suggest that several interventions may be beneficial for improving cervical cancer screening rates among immigrant women in Ontario. First, efforts need to be made to ensure that immigrant women get connected with the health care system after arrival and find a regular source of primary care. Settlement agencies may be able to play a substantial role toward this goal. Moving from Ontario's current system of opportunistic screening to one of centrally organized screening with periodic invitations may also be of benefit for increasing screening rates. Although it is neither feasible nor desirable for every immigrant woman to see a female provider, efforts should also be made to increase the enrolment of immigrant women in primary care patient enrolment models. Importantly, some primary care models may also make it feasible for male physicians to have female health professionals, such as trained nurses, physician assistants or nurse practitioners, available to provide cervical cancer screening, which may increase immigrant women's comfort with having the procedure performed. As well, targeted physician education campaigns for physicians trained abroad may be beneficial for improving screening rates. Future work should examine the reasons for lower screening rates when there is ethnic congruence between a physician and patient. Targeted patient education campaigns and interventions for all immigrant women will likely also be of utmost importance, with a particular focus on younger and older women, and on women of low income.

## Competing interests

The authors declare that they have no competing interests.

## Authors' contributions

AL took primary responsibility for the design of the study, the acquisition, analysis and interpretation of data, and drafting and revising the article. RM provided advice and direction for the study design and contributed substantially to data analysis and interpretation. SH provided advice and direction for the study design and contributed substantially to data interpretation. RG took primary responsibility for the conception of the study. He provided advice and direction for the study design, and data acquisition, analysis and interpretation. All authors revised the article critically for important intellectual content and gave final approval of the version to be published.

## Pre-publication history

The pre-publication history for this paper can be accessed here:

http://www.biomedcentral.com/1472-6874/11/20/prepub
